# Impact of Dietary Patterns and Serum Amino Acid Profile on Metabolic Syndrome Development in Mexican Women with Polycystic Ovary Syndrome

**DOI:** 10.3390/ijms252111821

**Published:** 2024-11-04

**Authors:** Midory Sánchez Rentería, Jorge Arturo Parra Montoya, Geraldine Sosa Romero, Lizbeth de Jesús González Piñuelas, Adriana M. López-Barradas, Omar Granados-Portillo, Mariel García Chagollán, Ana Laura Pereira Suárez, Patrick M. Gillevet, Natali Vega Magaña, Marcela Peña Rodríguez

**Affiliations:** 1 Master in Medical Microbiology, Centro Universitario de Ciencias de la Salud, Universidad de Guadalajara, Guadalajara 44340, Mexico; midory.sanchez9867@alumnos.udg.mx; 2 Servicio de Ginecología y Obstetricia, Hospital Civil Juan I. Menchaca, Guadalajara 44340, Mexico; jorgepama56@gmail.com (J.A.P.M.); geraldine.sosa94@hotmail.com (G.S.R.); 3 Laboratorio de Diagnóstico de Enfermedades Emergentes y Reemergentes, Departamento de Microbiología y Patología, Centro Universitario de Ciencias de la Salud, Universidad de Guadalajara, Guadalajara 44340, Mexico; lizgonalez_3291@hotmail.com (L.d.J.G.P.); alejandra.vega@academicos.udg.mx (N.V.M.); 4 Departamento de Fisiología de la Nutrición, Instituto Nacional de Ciencias Médicas y Nutrición Salvador Zubirán, Mexico City 14080, Mexico; adrimar24@gmail.com (A.M.L.-B.); ograpo@yahoo.com (O.G.-P.); 5 Instituto de Investigación de Ciencias Biomédicas, Centro Universitario de Ciencias de la Salud, Universidad de Guadalajara, Guadalajara 44340, Mexico; chagollan@academicos.udg.mx (M.G.C.); analauraps@hotmail.com (A.L.P.S.); 6 Microbiome Analysis Center, George Mason University, Manassas, VA 20110, USA; pgilleve@gmu.edu

**Keywords:** PCOS, metabolic syndrome, BCAA, diet, nutrition

## Abstract

Polycystic ovary syndrome (PCOS) is the main endocrine disorder in women of reproductive age worldwide. This condition is often associated with various metabolic alterations that contribute to the development of metabolic syndrome (MetS). Recent research suggests that branched-chain amino acid (BCAA) dysregulation is observed in PCOS. This study aims to investigate the relationship between dietary patterns, body composition, metabolic analytes, and serum amino acid levels in Mexican women with PCOS. Utilizing a cross-sectional design, we found that both study groups, PCOS (*n* = 24) and PCOS + MetS (*n* = 21), exhibited increased relative fat mass and dietary habits characterized by high simple sugar intake and low protein consumption, correlating with levels of relative fat mass and leptin. Notably, serum concentrations of BCAAs and glutamic acid were significantly elevated in the PCOS + MetS group. Our findings suggest that a metabolic approach may enhance the prediction and management of MetS in women with PCOS, highlighting the importance of dietary interventions in this population.

## 1. Introduction

Polycystic ovary syndrome (PCOS) is the main endocrine disorder that affects women of reproductive age, with a prevalence of between 5 and 20% worldwide [1,2]. It has been observed that PCOS can be accompanied by alterations, which together can influence the development of metabolic syndrome (MetS), such as insulin resistance (IR), hypertension, dyslipidemia, abdominal obesity, and an alteration of glucose metabolism [3,4]. Among them, IR, which occurs in 50 to 90% of women with PCOS, plays an important role as a mechanism associated with the presence of MetS, being a precursor of various alterations that lead to metabolic imbalance [5,6], while abdominal obesity, a phenotype usually observed in 50% of women with PCOS, is the main clinical predictor of MetS [7,8].

Among the factors involved in the presence of MetS, it is proposed that branched-chain amino acids (BCAAs)—valine, leucine, and isoleucine—could be involved, as a positive correlation with IR and obesity has been reported [9]. Being part of the group of essential amino acids, BCAAs are components that are mainly obtained from food, constituting approximately 20% of dietary proteins. Apart from being considered substrates for protein synthesis, their importance lies in their participation as signaling molecules in the regulation of glucose, lipid, and protein metabolism, in such a way that a disruption in their catabolism can lead to an increase in their levels in serum, which has been significantly associated with various metabolic disorders [10].

In women with PCOS, significantly high levels of BCCAs have been reported, both as separate amino acids and as a group, which could be established as a risk factor for the development of a metabolic imbalance and consequently the presence of MetS [11], which together would influence the severity of PCOS and the presence of other comorbidities [12]. Therefore, this study aimed to explore the relationship between diet, body composition, metabolic analytes, and serum amino acids in Mexican women with PCOS to elucidate possible elements that drive MetS in these patients.

## 2. Results

### 2.1. Anthropometric, Hormonal, and Nutritional Profiles

Anthropometric, hormonal, and nutritional profiles were evaluated from the PCOS and PCOS + MetS groups (Table 1). As expected, the PCOS + MetS group presented significantly higher values of weight, BMI, RFM, and WHR; nevertheless, exercise was found in a similar proportion between both groups.

**Figure 1:**
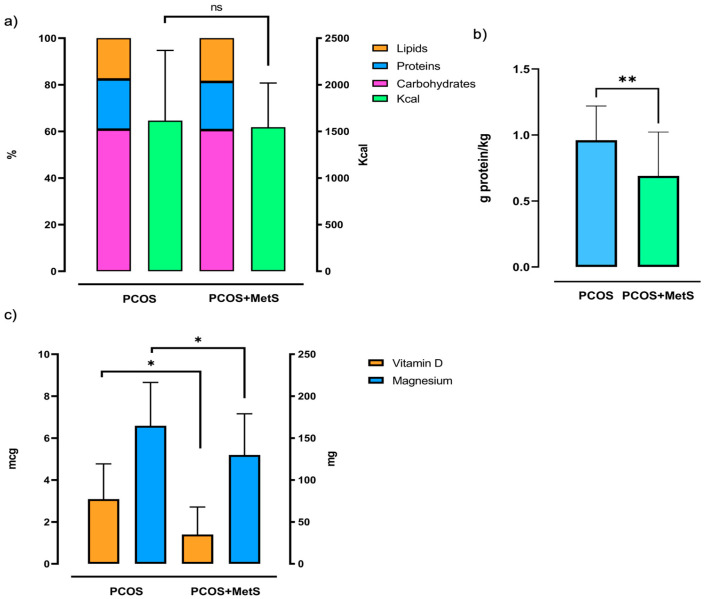
Nutrient intake comparison between PCOS and PCOS + MetS groups. (**a**) Percentage of macronutrient distribution in both group’s diet vs total caloric consumption, (**b**) protein intake by weight in kilograms, and (**c**) micronutrient intake of vitamin D (mcg) and magnesium (mg). Mann–Whitney U Test with median and interquartile range, * *p* < 0.05; ** *p* < 0.01; ns: not significant. PCOS: polycystic ovary syndrome, PCOS + MetS: polycystic ovary syndrome + metabolic syndrome, mcg: microgram, mg: milligram.

Furthermore, HOMA-IR, LH, and LH/FSH values were significantly higher in the PCOS + MetS group. In contrast, the PCOS group had a higher caloric intake, but a similar distribution of macronutrients consumed (Figure 1a). It is worth noting that women with PCOS + MetS had significantly lower grams of protein consumed per kg of weight compared with the PCOS group (Figure 1b), which is below the daily recommendation for the healthy population according to the US recommended dietary allowances [13]. According to the expert committee of the Food and Nutrition Board of the National Academies of Sciences, Engineering, and Medicine, the consumption of vitamin D and magnesium in both groups was below the daily recommended intake of 15 ug and 320 mg, respectively; this deficit was more pronounced in the PCOS + MetS group [14,15] (Figure 1c).

It is also worth noting that according to the Dietary Guidelines for Americans, both groups of the study reported an excess of sugar consumption (g) and saturated fats [16]. Additionally, all women had an omega-3 consumption below the adequate intake reported by the committee of the Food and Nutrition Board [17]. Furthermore, the recommended daily value of fiber is 28 g; this reflects a marked deficit in the consumption of these nutrients by the women in both groups [18].

### 2.2. Serum Amino Acid Profile

Serum amino acids were detected individually; subsequently, we classified them into BCAAs and aromatic amino acids (AAAs). The PCOS + MetS group had statistically higher levels of glutamic acid (*p* ≤ 0.001), threonine (*p* = 0.008), alanine (*p* = 0.003), tyrosine (*p* ≤ 0.001), valine (*p* = 0.035), tryptophan (*p* = 0.029), phenylalanine (*p* = 0.002), leucine (*p* = 0.001), lysine (*p* = 0.006), proline (*p* = 0.032), the BCAAs (*p* = 0.030), and the AAAs (*p* ≤ 0.001) (Table 2).

### 2.3. Metabolic Profile

**Figure 2:**
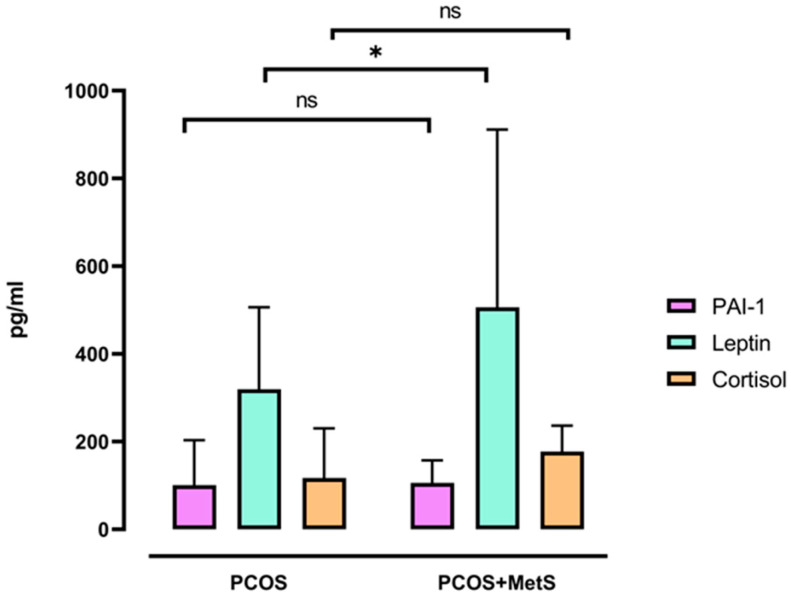
Metabolic profile evaluation of women with PCOS and PCOS + MetS. Serum levels of PAI-1, leptin, and cortisol are shown. Mann–Whitney U Test with median and interquartile range. * *p* < 0.05; ns: not significant. PCOS: polycystic ovary syndrome, PCOS + MetS: polycystic ovary syndrome + metabolic syndrome.

For the metabolic panel assay, plasminogen activator inhibitor 1 (PAI-1), leptin, and cortisol were evaluated. PAI-1 is a marker of IR as its levels are usually elevated in insulin-resistant states; therefore, patients with PCOS might show the same pattern. However, no difference was observed in PAI-1 between both groups. A significant difference was observed in leptin levels (*p* < 0.05), which were higher in the PCOS + MetS group. It is worth noting that this same group showed a tendency for higher cortisol values, but statistical significance was not obtained (Figure 2).

### 2.4. Identification and Correlation of Parameters Involved in the Development of Metabolic Syndrome in PCOS

**Figure 3:**
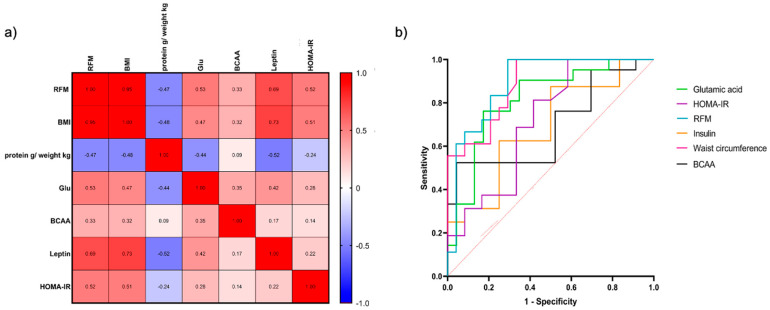
Identification of analytes and measurements involved in metabolic syndrome development in PCOS. (**a**) Spearman heatmap of anthropometric, nutritional, and metabolic parameters. (**b**) ROC curve of evaluated markers for metabolic syndrome prediction in women with PCOS. Darker blue indicates a negative correlation, and red indicates a positive correlation; a *p*-value < 0.05 was considered significant.

Non-parametric correlation analysis was performed to study the association between diet, body composition, and serum amino acids. The Spearman’s rank heatmap showed significant correlations from each profile analysis (Figure 3a). In the case of RFM and BMI, a negative correlation was identified with g/kg protein consumption (r = −0.47, *p* = 0.002; r = −0.48, *p* = 0.001, respectively) and a positive correlation with the levels of glutamic acid (r = 0.53, *p* ≤ 0.001; r = 0.47, *p* = 0.0018), BCAAs (r = 0.33, *p* = 0.035; r = 0.32, *p* = 0.045), leptin (r = 0.69, *p* ≤ 0.001; r = 0.73, *p* ≤ 0.001), and HOMA-IR (r = 0.52, *p* = 0.008; r = 0.51, *p* = 0.008). Interestingly, g/kg protein consumption was negatively correlated with the levels of leptin (r = −0.52, *p* = 0.001) and glutamic acid (r = −0.44, *p* = 0.004).

Furthermore, to predict MetS development in patients with PCOS, we carried out an area under the curve (AUC) analysis using different analytes and measures (Figure 3b). Three of these analytes and measures presented the highest sensitivity and specificity values for MetS prediction. RFM presented 83.33% sensitivity and 79.17% specificity; similarly, waist circumference and glutamic acid showed values of 72.2% and 76.19% sensitivity and 79.17% and 82.61% specificity, respectively. Based on these results, the cut-off values proposed for the RFM, waist circumference, and glutamic acid are 4, 3.46, and 4.38, respectively.

## 3. Discussion

The association of dietary intake with metabolic health status has long been recognized in humans; moreover, nutritional patterns that include micronutrients as well as body composition measures and other analytes can help leverage the interpretation of the information from a given population. In this study, a low-protein, high-sugar diet with poor intake of fiber, vitamin D, and magnesium characterized women with PCOS. These dietary patterns can impact other measures and analytes that can promote the occurrence of MetS in women with PCOS. In particular, women with PCOS have higher levels of BCAAs in their plasma compared with controls, and these values are higher in women with PCOS and MetS; therefore, this could be related to the metabolic disruption highly prevalent in this population.

After classifying our patients, most of the women with PCOS without MetS also showed insulin resistance by HOMA-IR, confirming the main metabolic disturbance widely reported in this endocrine pathology [19,20]. Insulin resistance has been related to BMI and other metabolic markers such as adipokines [21,22,23]. In this line, other metabolic analytes such as leptin have been proposed as predictive markers for PCOS [24] and metabolic syndrome separately [25]. Our study confirmed that leptin levels are significantly higher in women with MetS and PCOS. Altogether, this shows the importance of specific therapies to limit metabolic disorders in patients with PCOS.

Diet plays a main role in PCOS management and control; based on the insulin resistance state, most dietary interventions in PCOS involve limiting carbohydrate intake and a low glycemic load [26,27,28]. Hence, it is important to identify the dietary patterns of the population of the study. Here, we detected a high sugar intake by both groups of the study, with no difference in total carbohydrate consumption. Interestingly, protein intake by weight was below the recommended dietary allowance (RDA) of protein for a healthy adult [29] in the PCOS + MetS group, which also had a more marked deficiency in fiber, vitamin D, and magnesium intake compared with the PCOS group. This information emphasizes the value of analyzing protein intake by weight as well as other micronutrients and not only limiting carbohydrate intake in this population.

Dietary fiber is an important gut microbiome modulator as well as a positive regulator of glucose metabolism [30]. The recommended intake ranges from 25 to 30 g per day for a healthy adult [31]. In this study, both groups had less than 7 g of fiber consumption per day; this limitation can lead to shifts in the gut microbial metabolism toward the utilization of dietary and endogenously supplied proteins and host mucins [32]. Moreover, low fiber can also lead to increased metabolites derived from the fermentation of amino acids, including branched-chain fatty acids, ammonia, amines, and phenolic compounds [33]. It is clear that dietary fiber also plays a crucial role in PCOS and should be part of dietary recommendations in these patients.

Low fiber consumption can alter serum amino acid levels through gut microbiota modulation. The amino acid profile can be linked with metabolic disturbances; specifically, BCAAs are associated with insulin resistance, obesity, and even T2DM. Consistently, the PCOS + MetS group showed significantly higher values of BCAAs and AAAs, which has previously been reported in other populations with PCOS [34]. Even though the underlying mechanism has not been elucidated, gut microbiota might play an important role as PICRUSt analysis has shown increased synthesis in BCAAs [35]. Specific bacterial species have yet to be identified. Still, women with PCOS have shown a higher level of Lachnospiraceae compared to controls, and this was even identified as a causal link with PCOS [35,36]. Interestingly, this bacteria has metabolic pathways of aromatic amino acids involved in the biosynthesis of indole-propionic acid, indole, phenol, and p-cresol [37], which suggests a link between dietary fiber, gut microbiota, and serum amino acid profile in women with PCOS.

The mechanisms proposed for BCAA elevation in PCOS are abnormal degradation and higher production by gut microbiota. In this context, the positive correlation that we found of RFM with the amino acid glutamic acid was stronger than with the BCAAs. Other authors have also reported this and linked these alterations to metabolic syndrome risk [38]. Only glutamic acid but not the BCAAs showed a correlation with leptin and HOMA-IR, which can be linked to the fact that the TCA cycle and glucose metabolism are the major pathways altered in PCOS [39].

Intriguingly, RFM, waist circumference, and glutamic acid exhibited the best signature of MetS in Mexican women with PCOS, which shows the power of anthropometric evaluation and dietary assessment, as protein by weight intake was negatively associated with RFM, and biochemical measurements in this population should not be left aside. On the other hand, further investigation of amino acid dysregulation in PCOS, in particular glutamic acid and BCAAs, might aid not only in predicting metabolic disturbances but also in early PCOS diagnosis and as promising future pharmacological targets, as shown elsewhere [38,40].

Hormesis describes the beneficial effect of an adaptive response to a low dose of a stressor that in higher concentrations shows a harmful effect on the organism [41], where the specific reaction to this principle is called a hormetic response. When applied to dietary micronutrients that can elicit a hormetic response, these can be called hormetic nutrients; some examples are omega-3 fatty acids, polyphenols, plant or fungi extracts, fibers, glutamic acid, and vitamin D. Importantly, low-dosage responses result in enhanced stress resilience/adaptive capacity via anti-inflammatory and antioxidant molecular networks; therefore, an imbalance in the consumption or levels of these nutrients can impact the oxidative status of the cell [42].

Previous studies have reported that shifts in the oxidative status can contribute to the pathogenesis and prognosis of PCOS [43,44]. Nuclear factor erythroid 2-related factor 2 (Nrf2) is a key antioxidant pathway highly influenced by bioactive compounds that can be considered hormetic nutrients. Some of these nutrients include omega-3 fatty acids, polyphenols, and vitamin D, and they have gained attention for their potential to manage PCOS and the associated MetS [44,45,46]. This underscores the importance of research interventions based on personalized micronutrient recommendations and time-delimited food intake for PCOS and its related comorbidities. The synergistic effects of these dietary compounds should also be explored to unveil precision nutrition strategies tailored for individuals with PCOS.

Nevertheless, this study has several limitations that must be acknowledged. First, the cross-sectional design of the study limits the ability to infer causal relationships between dietary intake, amino acid profiles, and the development of metabolic syndrome. Longitudinal studies would be needed to establish causal links. Second, dietary intake was assessed through self-reported questionnaires, which are subject to recall bias and inaccuracies in reporting, potentially affecting the reliability of the dietary data. Third, the sample size, although adequate for the statistical analyses, may not be large enough to fully capture the diversity of dietary patterns and metabolic outcomes across different subgroups of Mexican women with PCOS.

## 4. Conclusions

In this cross-sectional study, both groups of women showed elevated adiposity by relative fat mass and a diet characterized by a higher intake of simple sugars and low protein consumption, and these dietary patterns correlated with RFM and leptin levels. Importantly, serum amino acids, in particular glutamic acid and BCAAs, were significantly elevated in the PCOS + MetS group. A metabolic approach may aid in prediction and treatment to prevent metabolic syndrome development in patients with PCOS.

## 5. Materials and Methods

### 5.1. Study Design and Setting

In this cross-sectional study, 45 women with PCOS aged 15 to 45 years old and from the Gynecology and Obstetrics service of the Dr. Juan y Menchaca Civil Hospital were recruited from August 2023 to August 2024 and separated into two groups: polycystic ovary syndrome (PCOS *n* = 24) and PCOS with metabolic syndrome (PCOS + MetS *n* = 21). PCOS diagnosis was confirmed according to the 2006 Androgen Excess Society Guide and MetS with the Adult Treatment Panel III criteria of the National Cholesterol Education Program (NCPE). Patients who had elevated prolactin levels; thyroid disease or Cushing’s disease; previous ovarian surgery; used antibiotics, prebiotics, probiotics, symbiotics, or laxatives two months before recruitment; used pharmacological agents or hormones that could affect the course of the menstrual cycle or metabolism three months before recruitment; used weight loss supplements; a clinical diagnosis of gastrointestinal disorders; or were pregnant or breastfeeding were excluded.

Written informed consent was obtained from all patients. In the case of underage patients, informed consent was provided by the parent or guardian. Ethical approval was obtained from the hospital and the Centro Universitario de Ciencias de la Salud Ethics Committee with the registration numbers 00041 and CI-06723, respectively. After signing, patients had a clinical, biochemical, nutritional, and anthropometric evaluation.

### 5.2. Anthropometric and Nutritional Assessment

Body composition was determined by an ISAK level 3, with anthropometric measurements taken according to the restricted ISAK protocol: weight (Tanita Rd-545im Ironman, Tokyo, Japan), height (SECA, 213, Hamburg, Germany), mid-arm muscle area, contracted arm, waist, abdomen, hip, thigh, and leg. All circumferences were obtained using a Lufkin W606PM anthropometric tape (Missouri City, TX, USA). Eating habits were assessed using a 24-hour recall on 3 alternate days, which were analyzed using ESHA’s Food Processor^®^ Nutrition Analysis software v 11.1 by a certified dietitian.

### 5.3. Metabolic and Hormonal Profile Analysis

Peripheral blood samples were collected from all subjects during days 2–3 of spontaneous cycles after an overnight fast. Fasting glucose, insulin, total testosterone, estradiol, follicle-stimulating hormone (FSH), and luteinizing hormone (LH) levels were measured by the hospital laboratory. The insulin resistance index (HOMA-IR) was calculated using homeostasis model assessment methods, defined as fasting serum glucose (mg/dL) × fasting insulin (mIU/L)/405.13.

Additionally, a metabolic panel was performed using a pearl immune assay multiplex (Human Diabesity Panel [4-plex], LegendPlex cat. 740913, Biolegend, San Diego, CA, USA) following the manufacturer’s instructions and by reading samples in an Attune NXT Flow Cytometer (Thermo Fisher Scientific, Waltham, MA, USA).

### 5.4. Amino Acid Profile Determination

High-Performance Liquid Chromatography was employed for amino acid level determination in serum samples. First, 150 μL of plasma was added to 38 μL of 10 % sulfosalicylic acid to deproteinize the sample. The samples were then incubated for 30 min at 4 °C and centrifuged at 14 000 rpm for 10 min to separate the supernatant. Then, 100 μL of the supernatant was taken, 1 μL of the internal standard (norvaline; 15 mM) was added, and the sample was derivatized and injected. The procedure was performed using a sampling device (Agilent; G1367F, Santa Clara, CA, USA) coupled to an HPLC system (Agilent 1260 Infiniti) and a fluorescence detector (Agilent; G1321B). A ZORBAX Eclipse AAA column (Agilent) was used and maintained at 40 °C. Chromatographic conditions were maintained according to the column’s technical instructions.

### 5.5. Statistics

Statistical analysis was performed using the IBM SPSS Statistics 27 program. Data are expressed as median and interquartile range in the case of continuous variables and compared using the Mann–Whitney U test; nominal variables are expressed as frequency and percentage and analyzed using the Chi-square test.

## Figures and Tables

**Table 1:** Anthropometric, hormonal, and nutritional profiles.

	PCOS (*n* = 24) Median (IQR)	PCOS + MetS (*n* = 21) Median (IQR)	TOTAL (*n* = 45) Median (IQR)	*p*
Age (years)	21.50 (5)	23 (9)	23 (8)	0.379
**Anthropometric Profile**				
Weight (kg)	70.12 (16.76)	89.35 (28.57)	78.67 (24.92)	<0.001 *
BMI (kg/m^2^)	27.51 (9.95)	35.97 (6.90)	31.29 (10.05)	<0.001 *
RFM (%)	36.05 (11.37)	43.99 (3.88)	40.78 (9.43)	<0.001 *
WHR	0.74 (0.07)	0.84 (0.09)	0.78 (0.10)	<0.001 *
Waist circumference	78.40 (19.72)	102.37 (18.59)	88.92 (21.64)	<0.001 *
Exercise				0.923
YES	9 (39.13%)	9 (45.00%)	18 (41.86%)	
NO	14 (60.87%)	11 (55.00%)	25 (58.13%)	
**Hormonal Profile**				
HOMA-IR	3.16 (1.73)	3.97 (4.18)	3.79 (2.20)	0.053
Insulin	14.66 (6.76)	17.72 (24.98)	16.37 (8.59)	0.159
Estradiol (pg/mL)	56 (46.75)	49.32 (63.34)	54.14 (45.14)	0.512
Testosterone (ng/mL)	0.46 (0.31)	0.52 (0.33)	0.48 (0.32)	0.861
LH (mUi/mL)	5.83 (3.50)	7.15 (4.99)	6.82 (4.58)	0.583
FSH (mUi/mL)	4.46 (3.07)	4.22 (1.84)	4.33 (2.61)	0.403
LH/FSH	1.65 (1.26)	1.97 (0.72)	1.83 (0.94)	0.462
**Nutritional Profile**				
Carbohydrates (g)	195.11 (121.85)	189.95 (107.75)	194.95 (107.66)	0.527
Sugars (g)	72.04 (29.45)	63.25 (25.44)	69.28 (27.51)	0.644
Proteins (g)	69.49 (24.55)	67.73 (37.48)	69.35 (27.88)	0.284
Lipids (g)	58.42 (32.01)	58.01 (42.49)	58.42 (32.54)	0.733
Saturated fats (g)	21.45 (11.62)	21.17 (14.22)	21.24 (11.73)	0.961
Polyunsaturated fats (g)	8.69 (7.42)	7.81 (7.13)	8.63 (6.91)	0.465
Omega-3 (g)	0.88 (0.87)	0.80 (0.87)	0.85 (0.87)	0.652
Omega-6 (g)	6.46 (4.20)	6.64 (5.88)	6.59 (5.02)	0.990
Fiber (g)	6.87 (8.45)	6.41 (5.37)	6.87 (7.51)	0.263

The data were analyzed by Mann–Whitney U test and are expressed as median with interquartile range (IQR). The qualitative data were represented by frequency and percentage and were analyzed by Chi-square test. * Indicates statistical significance; *p* ≤ 0.05. BMI: body mass index, RFM: relative fat mass, WHR: waist–hip ratio, HOMA-IR: Homeostatic Model Assessment for Insulin, LH: lieutenant hormone, FSH: follicle-stimulating hormone.

**Table 2:** Profile of total, branched-chain, and aromatic amino acids.

	PCOS (*n* = 23) Median (IQR)	PCOS + MetS (*n* = 21) Median (IQR)	TOTAL (*n* = 44) Median (IQR)	*p*
Asp	14.68 (4.40)	17.20 (11.47)	15.64 (7.80)	0.053
Glu	42.79 (16.67)	65.28 (35.91)	52.38 (37.67)	<0.001 *
Asn	33.95 (8.57)	37.29 (8.64)	35.42 (9.19)	0.296
Ser	108.70 (26.23)	115.42 (29.09)	112.32 (24.99)	0.488
Gln	471.08 (56.44)	502.79 (90.44)	475.52 (75.30)	0.307
His	61.88 (8.98)	62.01 (8.41)	61.94 (8.81)	0.411
Gly	233.25 (115.89)	203.54 (66.29)	209.55 (78.28)	0.200
Thr	93.42 (55.44)	130.21 (48.91)	110.05 (57.45)	0.008 *
Arg	89.18 (18.68)	97.62 (19.72)	93.42 (20.55)	0.222
Ala	385.72 (77.02)	484.98 (200.83)	431.47 (147.76)	0.003 *
Tyr	57.71 (17.73)	67.60 (21.19)	62.15 (19.24)	<0.001 *
Cys	225.40 (83.41)	215.23 (90.97)	222.25 (89.40)	0.769
Val	167.66 (30.34)	186.92 (43.97)	175.03 (40.06)	0.035 *
Met	14.61 (4.78)	17.57 (7.37)	15.56 (5.83)	0.059
Trp	40.02 (6.85)	44.65 (6.44)	41.40 (7.51)	0.029 *
Phe	53.02 (8.22)	62.11 (16.23)	57.35 (13.26)	0.002 *
Ile	54.28 (16.38)	58.45 (22.85)	55.13 (15.95)	0.084
Leu	103.47 (16.64)	116.69 (26.30)	105.98 (21.77)	0.001 *
Lys	199.44 (34.26)	225.06 (29.25)	220.13 (41.46)	0.006 *
Pro	200.96 (54.84)	271.10 (119.88)	215.24 (112.86)	0.032 *
BCAAs	336.30 (72.30)	369.57 (106.27)	337.41 (66.38)	0.030 *
AAAs	150.06 (15.59)	184.90 (39.28)	158.67 (38.85)	<0.001 *
BCAAs/AAAs	2.09 (0.39)	2.04 (0.43)	2.06 (0.37)	0.378

The data were analyzed by Mann–Whitney U test and are expressed as median with interquartile range (IQR). * Indicates statistical significance; *p* < 0.05 was considered statistically significant. Asp: aspartic acid, Glu: glutamic acid, Asn: asparagine, Ser: serine, Gln: glutamine, His: histidine, Gly: glycine, Thr: threonine, Cys: cysteine, Val: valine, Met: methionine, Trp: tryptophane, Phe: phenylalanine, Ile: isoleucine, Leu: leucine, Lys: lysine, Pro: proline, BCAAs: branched-chain amino acids, AAAs: aromatic amino acids.

## Data Availability

The raw data supporting the conclusions of this article will be made available by the authors on request. The datasets presented in this article are not readily available because the data are part of an ongoing study. Requests to access the datasets should be directed to Ph.D. Marcela Peña Rodríguez.
